# Operating Conditions Optimization via the Taguchi Method to Remove Colloidal Substances from Recycled Paper and Cardboard Production Wastewater

**DOI:** 10.3390/membranes10080170

**Published:** 2020-07-29

**Authors:** Mayko Rannany S. Sousa, Jaime Lora-García, María-Fernanda López-Pérez, Asunción Santafé-Moros, José M. Gozálvez-Zafrilla

**Affiliations:** Research Institute for Industrial, Radiophysical and Environmental Safety (ISIRYM) Universitat Politècnica de València (UPV), Plaza Ferrándiz y Carbonell s/n, 03801 Alcoy, Spain; maysanso@doctor.upv.es (M.R.S.S.); jlora@iqn.upv.es (J.L.-G.); assanmo@iqn.upv.es (A.S.-M.); jmgz@iqn.upv.es (J.M.G.-Z.)

**Keywords:** paper mill treated effluent, ultrafiltration, optimization, fouling, DoE, Taguchi method

## Abstract

Optimization of the ultrafiltration (UF) process to remove colloidal substances from a paper mill’s treated effluent was investigated in this study. The effects of four operating parameters in a UF system (transmembrane pressure (TMP), cross-flow velocity (CFV), temperature and molecular weight cut-off (MWCO)) on the average permeate flux (*J_v_*), organic matter chemical oxygen demand (COD) rejection rate and the cumulative flux decline (SFD), was investigated by robust experimental design using the Taguchi method. Analysis of variance (ANOVA) for an L_9_ orthogonal array were used to determine the significance of the individual factors, that is to say, to determine which factor has more and which less influence over the UF response variables. Analysis of the percentage contribution (P%) indicated that the TMP and MWCO have the greatest contribution to the average permeate flux and SFD. In the case of the COD rejection rate, the results showed that MWCO has the highest contribution followed by CFV. The Taguchi method and the utility concept were employed to optimize the multiple response variables. The optimal conditions were found to be 2.0 bar of transmembrane pressure, 1.041 m/s of the cross-flow velocity, 15 °C of the temperature, and 100 kDa MWCO. The validation experiments under the optimal conditions achieved *J_v_*, COD rejection rate and SFD results of 81.15 L·m^−2^·h^−1^, 43.90% and 6.01, respectively. Additionally, SST and turbidity decreased by about 99% and 99.5%, respectively, and reduction in particle size from around 458–1281 nm to 12.71–24.36 nm was achieved. The field-emission scanning electron microscopy images under optimal conditions showed that membrane fouling takes place at the highest rate in the first 30 min of UF. The results demonstrate the validity of the approach of using the Taguchi method and utility concept to obtain the optimal membrane conditions for the wastewater treatment using a reduced number of experiments.

## 1. Introduction

The pulp and paper (P & P) industry is ranked as the world’s third largest consumer of fresh water [1] and an important producer of wastewater with different organic and inorganic contaminants. Depending on the type of processes used in paper manufacture, the integration between production and environmental protection is one of the key topics in the paper industry.

According to the Confederation of European Paper Industries (CEPI) [2], Europe is the second largest producer of paper and paperboard with 22.7% (91.39 million tons) of world production, making it one of the most important industries in the European economic sector. The paper industries hold an important place in Spanish economy, as Spain is one of the European leaders in paper recycling, with 84% of the raw materials used by the paper industry containing recovered paper [2]. However, we cannot forget that water is, also, an essential raw material for manufacturing paper and paperboard, and effluent treatment is a critical part of the process [3]. In order to minimize the amount of freshwater used and the volume of effluent discharged, the European Commission has described the best available techniques to be adopted by the P & P industry [4].

A number of conventional processes have previously been used to treat the different types of paper mill wastewater including coagulation and flocculation [5], adsorption [6,7], advanced oxidation [8,9] and membrane filtration [10,11,12,13]. It is important to mention that paper mills have their own wastewater treatment plants but some water treatment methods typically used for the P & P are not environmentally efficient: e.g.: coagulation/flocculation using inorganic coagulants create disposal problems and conventional aerobic processes have not been efficient in the removal of color or recalcitrant compounds [14,15]. This inadequacy can also make it impossible to reuse water in the papermaking process. Therefore, factories must improve their treatment plants to achieve the pollutant loadings permissible under current regulations and/or to reuse their process water.

Membrane separation technology has been attracting increasing attention as an alternative method for the post-treatment of paper mill wastewater. Some processing methods, such as nanofiltration, ultrafiltration (UF) and reverse osmosis, have recently been used in paper mills to purify secondary and tertiary effluents using external biological treatment [7,16,17,18]. Major advantages of membrane separation processes are their scalability, low installation costs and easiness of operation. However, their technical and economic liability must be carefully assessed for each specific process.

Ultrafiltration is an attractive process for paper mill wastewater treatment and it can be used as an advanced tertiary treatment to remove suspended solids and dissolved and colloidal substances (DCS) during the treatment of paper industry effluent in order to facilitate the reuse of the treated wastewater and reduce fresh water consumption [19,20]. What makes it so attractive is that most of the pollutants consist of high-molecular-weight compounds and these are easily removed by UF [10,21].

However, membrane fouling is still a limiting factor for the adoption and use of UF on a large scale in paper manufacturing applications. This fouling results in a sharp decline in permeate flux and, thus, changes in membranes selectivity [10,22,23]. Membrane fouling also increases the process cost due to repeated plant shutdowns to clean and wash the membranes [24]. Previous studies have shown that the main foulants of the membranes used for paper industry wastewater are DCS including fatty acids, resin acids, lignins and trace amounts of sterols, steryl esters and triglycerides [19,25]. Currently, this treatment technology can only be used to filter paperboard mill effluent that has been pre-treated and that still does not meet discharge standards [26].

Statistical experimental design incorporating design of experiments (DoE) techniques can be used to investigate the effects of all the possible interactions between the factors at one time, while undertaking the fewest possible experiments. A review of the literature revealed that an increasing number of studies are being conducted using DoE approaches in the membrane technology field to optimize operating conditions [10,27,28,29,30,31,32]. The DoE approaches for robust design include the Taguchi method that combines mathematical and statistical techniques to arrive at a special design of experiments with an orthogonal array (OA) to study multiple factors with a small number of experiments. This saves time and money by reducing the number of experiments required in the investigation [16]. It is worth mentioning that this approach is becoming popular because it is easy to adopt and applies an efficient method for optimizing the operating parameters. 

This approach also allows studying the influence of each individual factor on the response variables, as well as on the effects of interactions between factors over the response variables, that is to say, all operational conditions varying simultaneously according to design array. This permits the factors that have the greatest and least influence to be determined, along with the optimal level for each factor in an OA [33]. In addition, in many UF approaches it is necessary to consider the application of multiple response optimization, because the process performance is often evaluated using several quality characteristics (responses). In this case, the Taguchi method and utility concept are useful tools for optimizing operating parameters in multiple characteristics responses [34].

The statistical analysis of variance (ANOVA) can be used to provide information on whether the operating parameters (factors) are statistically significant or not, as well as to identify the influence of individual factors and establish the relationships between the factors and operating conditions. In this analysis, the *p*-value index is used to know which operating parameters have a significant effect on the response variables. This information is complemented with the F-test which helps to identify whether or not each factor is significant at the selected confidence level [35,36]. In this study, ANOVA was also used to analyze the experimental results.

The aim of this work was to determinate the effect of operating conditions such as transmembrane pressure (TMP), cross-flow velocity (CFV), temperature and molecular weight cut-off (MWCO) on the average permeate flux, chemical oxygen demand (COD) rejection rate and cumulative flux decline (SFD), in addition to determining the optimal conditions for the given sets of values and to find the best response variables by using Taguchi experimental design and the utility concept. The results of this study may be used as a guideline when operating UF systems under the best conditions in a wastewater treatment plant (WWTP) in a papermaking factory. The filtration results and analysis of the experimental data presented and discussed in this study were carried out by using ANOVA to find the significance of the controlling factors and optimized using the Taguchi method to find the optimal operating conditions. A standard L_9_ orthogonal array was selected for experimental planning with four factors and three levels for each factor.

## 2. Materials and Methods

### 2.1. Paperboard Mill Treated Effluent Feedstock

The paperboard mill treated effluent (PMTE) used in this work came from a secondary clarifier effluent from a WWTP in a papermaking factory located in the south of the Valencian autonomous region in Spain. In order to prevent early membrane fouling, remove large suspended solids, and reduce initial turbidity and COD in the PMTE, the raw feed solution was pre-filtered by conventional filtration (low-pressure pump at around 1 bar) with a Cintropur^®®^ NW 50 filter element, and centrifugal propeller and filter cloths with a 5 μm nominal pore size. The significant characteristics of the PMTE samples are listed in Table 1.

### 2.2. Membranes and Experimental Setup

This study used polyethersulfone (PES) membranes provided by Synder Filtration™ (Vacaville, CA, USA) with MWCO of 10, 50 and 100 kDa, denoted 10-ST, 50-MQ and 100-LY, respectively.

The experiments were performed in a typical UF pilot plant with a flat-sheet membrane module (Rhône-Poulenc, Lyon, France), that allowed working with two membranes with similar or different MWCO (depending on the experiment being carried out). The effective area for each membrane in the module was 154.8 cm^2^. The details of the experimental set-up have been described previously by Sousa et al. [21]. The pilot plant had a data acquisition system (temperature, module input and output pressure) from LabVIEW^®^ (National Instruments, Austin, TX, USA). The permeate was collected during the filtration in a beaker placed on an electronic balance connected to a computer in order to continuously register the weighting data. This data was then automatically logged every 30 s and subsequently used to calculate permeate flux through the membranes.

### 2.3. Analytical Methods

The PMTE used as the feed solution and the UF permeate samples were analyzed according to the methods described below. The suspended solids analyses were carried out in accordance with the standard methods [37]. Turbidity was measured using a Dinko 112 turbidimeter (ASTM D1889, Barcelona, Spain). Conductivity was measured using a WTW level 3 conductivity device (ASTM D1125–14, Weilheim in Oberbayern, Germany). COD and total nitrogen in the effluent were analyzed using a Merck photometer (Merck KGaA, Darmstadt, Germany) and a Merck TR-300 thermoreactor (Merck KGaA, Darmstadt, Germany) in accordance with the standard methods.

### 2.4. Field-Emission Scanning Electron Microscopy (FESEM)

Field-emission scanning electron microscopy (FESEM) measurements were used to provide information on the fouling that formed on the membranes. The surface and cross-section morphologies of the fresh and fouled membranes were observed by field-emission scanning electron microscope, (ZEISS ULTRA 55, Oxford Instruments, Berkshire, UK), operated with a voltage of 200 kV and an accelerating voltage of 0.02–5 kV. Before analysis, the dried membrane samples were attached to double-sided adhesive carbon tape on an aluminum holder, and subsequently coated with a thin layer of gold prior to analysis.

### 2.5. Experimental Procedure

#### 2.5.1. Membrane Characterization

Before the UF runs, permeability experiments were carried out to determine the intrinsic membrane resistance (*R_m_*). Distilled water was used as the feed solution and measurements were taken for 1.0, 1.5, 2.0, and 3.0 bar of transmembrane pressure (*TMP*) at 1.041 m/s and 22.5 °C, in total recirculation mode to generate a quasi-steady state. The characterization process was undertaken for an operation time of 2 h to stabilize the flux through the membrane during this time. Previously each membrane was worked under compaction conditions with pure water at 5 bar for 1 h, in order to obtain a stable membrane structure. *R_m_* values were calculated using the resistance model (Equation (1)), where under this condition, there was no membrane fouling resistance (*R_f_*) [38]:

(1) $$J_{P}=\frac{TMP}{\mu \;R_{t}}=\frac{TMP}{\mu \;\left(R_{m}+R_{f}\right)}$$

where *J_P_* is the permeate flux, *μ* is the viscosity of the permeate stream and *R_t_* is the total membrane resistance. *R_f_* can be understood as the result of the sum of the three main fouling mechanisms: (*i*) pore blockage resistance when colloids block the membrane pores, (*ii*) adsorption resistance as a result of foulant adsorption inside or over the membrane and, (*iii*) cake layer resistance as a consequence of the accumulation of particles on the membrane [35,39,40].

#### 2.5.2. Ultrafiltration Experiments

The UF experiments were performed in crossflow filtration mode. The studied parameters were varied in the following ranges: TMP (1.0–3.0 bar), CFV (0.463–1.041 m/s), MWCO (10–100 kDa) and temperature (15–30 °C). These values were selected based on the operational limits of the experimental setup, industrial scale-up and economic considerations [21].

The evolution of permeate flux was gravimetrically measured at different time intervals according to Equation (2),

(2) $$J_{P}=\frac{1}{A_{m}\;\rho }\frac{dm_{p}}{dt}$$

where *A_m_* is the effective membrane area, *m_p_* is the total mass of permeate, *ρ* is the water density, and *t* is the filtration time.

In order to keep the feed concentration constant, both the permeate and the retained streams were continuously recirculated to the feed tank.

To evaluate the UF performance in terms of permeability the average permeate flux was calculated by the following equation [41]:

(3) $${\bar{J}}_{P}=\frac{1}{t_{M}}\int_{t_{1}}^{t_{M}}J_{P}\left(t\right)\cdot dt$$

where *J_P_*(*t*) is the permeate flux evolution over time determined by regression analysis on the experimental data, *t*_1_ is the initial time operation (first data collected), and *t_M_* is 2 h.

To analyze the effect of the operating conditions on UF resistance, the cumulative flux decline (SFD) [41] was calculated from the following equation:

(4) $$SFD=\overset{M}{\underset{i=1}{\sum }}\frac{J_{P}\left(0\right)-J_{P}\left(i\right)}{J_{P}\left(0\right)}$$

where *M* is the number of experimental data collected and, *J_P_* (0) is the initial permeate flux measured at $t_{1}$.

This parameter gives information about how the flux declines over the duration of the experiment (not just the difference between the initial and final permeate flux). Therefore, the higher the *SFD* value, the faster and more noticeable is the flux decline, indicating that the membrane fouling is more severe.

The average flux decline index ($\bar{FD})$ provides information on the decrease of feed permeate flux throughout the experiment estimated as follows:

(5) $$\bar{FD}=\left[\frac{1}{t_{M}}\int_{t_{1}}^{t_{M}}\frac{J_{P}\left(0\right)-J_{P}\left(t\right)}{J_{P}\left(0\right)}dt\right]\times 100$$

To evaluate the UF efficiency in removing organic matter, the COD rejection rate was chosen as the response variable calculated as:

(6) $$R\left(\%\right)=\left(1-\frac{C_{p}}{C_{f}}\right)\times 100$$

where: *C_p_* and *C_f_* are the COD concentration in the permeate and feed, respectively.

### 2.6. Experimental Design Based on the Taguchi Method

An experimental design based on the Taguchi method was used to design the experiments. The Taguchi method applies fractional experimental designs, called orthogonal arrays (OA), to reduce the number of experiments required to determine the optimum conditions based on the results [29,30,42]. One of the important steps in the Taguchi approach is the appropriate selection of OAs, which depends on the number of control factors and their levels. The minimum number of experimental trails required in an OA is given by *N_min_* = *(L* − 1*)·F* + 1, where *F* and *N* are the number of factors and levels respectively [36,43].

As mentioned above, the four factors (parameters) chosen were the transmembrane pressure, the cross-flow velocity, the temperature and the MWCO of the membrane; and three response variables were analyzed: the average permeate flux, the COD rejection and the cumulative flux decline. The selected factors, their designated symbols and levels are presented in Table 2.

For an experimental design with four factors and three levels for each factor, an L_9_ (3^4^) orthogonal array was selected. In this case, 27 runs were conducted (three repetitions at each trial condition). The design of the experiments planning matrix for the L_9_ array [43] is shown in Table 3. The experiments were carried out in a randomized order. New membrane samples were preconditioned and used in the experimental runs (27 membrane samples). In this way, the lurking effect of possible irreversible fouling was avoided and the intrinsic variability of the membrane material is included in the replications.

The aim of this DoE was to determine the operating parameters (factors) under which the average permeate flux and COD rejection rate achieve their maximum values, and the SFD achieved its minimum value. The Taguchi method uses a statistical measure of the process performance, called signal-to-noise ratio (S/N), which depends on the criterion for the response variable to be optimized. The S/N ratios are divided into three different categories and data sets, the larger-the better, the smaller-the-better and the nominal-the-better [16,29]. In this study, the system was optimized when the average permeate flux and COD rejection rate were as large as possible (Equation (7)), and the SFD was as small as possible (Equation (8)):

(7) $$\mathrm{The}\;\mathrm{larger}-\mathrm{the}-\mathrm{better}\;(S/N)=-10\;\log\left(\frac{1}{n}\overset{n}{\underset{i=1}{\sum }}\frac{1}{Y_{i}^{2}}\right)$$

(8) $$\mathrm{The}\;\mathrm{smaller}-\mathrm{the}-\mathrm{better}\;\left(S/N\right)=-10\;\log\left(\frac{1}{n}\overset{n}{\underset{i=1}{\sum }}Y_{i}^{2}\right)$$

where *Y_i_* the response variable at each experiment and *n* is the total number of repetitions in a trial.

**Figure 1:**
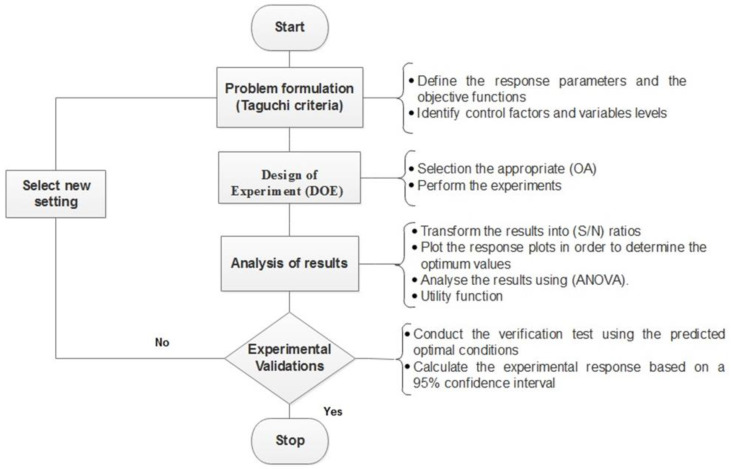
Flow diagram of Taguchi method steps to optimize a UF process to remove dissolved and colloidal substances (DCS) from paperboard mill treated effluent [43,44].

The sequence of steps to be followed using the Taguchi method to optimize the UF process is shown in Figure 1.

Minitab Statistical and Statgraphics Centurion XVII Software were used to analyze the Taguchi experiments and optimize the operating conditions.

#### 2.6.1. Utility Concept

The implementation of the utility concept in the Taguchi method helps to obtain the best combination of operating parameters to optimize multiple response S/N ratios (MRSN) simultaneously by differentiating the relative importance (weights) of various responses [34,45,46]. In this work, it is assumed that the overall utility is the sum of the responses of the individual utilities and it can be written as [34,47]:

(9) $$U\left(x_{1},x_{2,}\ldots x_{n}\right)=f\;\left[U_{1}\left(x_{1}\right),U_{2}\left(x_{2}\right),\ldots U_{n}\left(x_{n}\right)\right]$$

where *U*(*x*_1_,*x*_2_,…*x_n_*) is the overall utility of *n* response variables and *U_i_*(*x_i_*) is the utility index of *i^th^* response.

The response variables can be attributed priorities depending upon the process goals to be achieved. The priorities can be adjusted by providing a weight to the individual utility index. Therefore, by assigning weights to the response variables, the overall utility function can be expressed as:

(10) $$U\left(x_{1},x_{2,}\ldots x_{n}\right)=\overset{n}{\underset{i=1}{\sum }}W_{i}U_{i}\left(x_{i}\right)$$

where *W_i_* is the weight assigned to the *i^th^* response variable.

It is worth noting that the assignment of weights is a purely subjective (empirical) step and depends on each experiment or process that will be carried out [48]. Therefore, in this paper, the analytic hierarchy process (AHP) method, developed by [49] was used to determine the associate weight criteria for each response variable in the multiple optimization required to calculate the overall utility index. The relative normalized weight *W_i_* of each criterion is calculated using the AHP geometric mean method *GM_i_* on the rows in the pairwise comparison matrix, A║*a_ij_*║and it can be calculated from the follow equation [49]:

(11) $$W_{i}=\frac{GM_{i}}{\sum_{i=1}^{M}GM_{i}}$$

where *i,j* = 1, 2, …, *M* and *M* is the number of factors in judgement matrix A.

*a_ij_* = 1 for *i = j*,

*a_ij_* = 1/*a_ij_* for *i ≠ j*.

In addition, the total sum of the weight for all the responses must be assigned to hold the following condition:

(12) $$\overset{n}{\underset{i=1}{\sum }}W_{i}=1$$

For this study, as stated above, the objective was to maximize permeate flux and COD rejection rate, and minimize the SFD, simultaneously. From the utility concept, the MRSN of the overall utility value is given by Equation (13):

(13) $$\mu_{MRSN}=W_{{\bar{J}}_{P}}\mu_{1}+W_{COD\_rejection\;}\mu_{2}+W_{SFD}\mu_{3}$$

where:

(14) $$\mu_{1}=-10\log\left(\frac{1}{J_{\bar{P}}{}^{2}}\right)$$

(15) $$\mu_{2}=-10\log\left(\frac{1}{COD_{rejection}{}^{2}}\right)$$

(16) $$\mu_{3}=-10\log\left(SFD^{2}\right)$$

*W_JP_*, *W_COD_rejection_*, and *W_SFD_* are the weights assigned to the permeate flux, COD rejection rate and SFD.

It is worth mentioning that the utility function is of the higher-the-better type. If the composite measure (the overall utility) is maximized, the quality characteristics considered for the evaluation of utility will automatically be optimized (maximized or minimized) [44].

#### 2.6.2. Optimal Performance Prediction

Once the optimal level of the operating conditions has been selected, it is possible to predict and verify the utility responses using the optimal parameters. The predicted response values under optimal conditions *Y*_opt_ can be calculated from Equation (17) [50,51]:

(17) $$Y_{\mathrm{opt}}=m+\overset{p}{\underset{j=1}{\sum }}\left[\left(m_{i,j}\right)-m\right]$$

where *m* is the overall mean value of $\mu_{\mathrm{MRSN}}$ over nine trials; *m_i,j_* is the mean value of the quality response under optimal conditions; and *p* is the number of significant operating parameters that affect the UF process.

The 95% confidence interval for the confirmation experiments (CI_CE_) must be evaluated at the selected error level according to the following expression [43,50,51]:

(18) $${\mathrm{CI}}_{\mathrm{CE}}=\pm \sqrt{F_{\alpha }\left(1,f_{e}\right)\cdot MS_{e}\left(\frac{1}{n_{eff}}+\frac{1}{R}\right)}$$

where *F_α_* (1,*f_e_*) is the F-ratio at a confidence level of (1 − *α*) against a DOF equal to one and an error degree of freedom *f_e_* and, *n_eff_* is the effective sample size calculated as:

(19) $$n_{eff}=\frac{N}{1+\left(\mathrm{DOF}\;\mathrm{of}\;\mathrm{all}\;\mathrm{factors}\;\mathrm{used}\;\mathrm{to}\;\mathrm{estimate}\;\mathrm{the}\;\mathrm{mean}\right)}$$

where *N* is the number of experiments, *R* is the number of repetitions, and *MS_e_* is the error variance.

### 2.7. Analysis of Variance (ANOVA)

In order to determine the relative importance of the factors, ANOVA was employed by calculating the sum of squares (*SS*), degrees of freedom (*DOF*), mean of square (*MS*), associated F-test of significance (*F*) and percentage contribution (*P%*) [52].

## 3. Results and Discussions

### 3.1. Experimental Results

**Figure 2:**
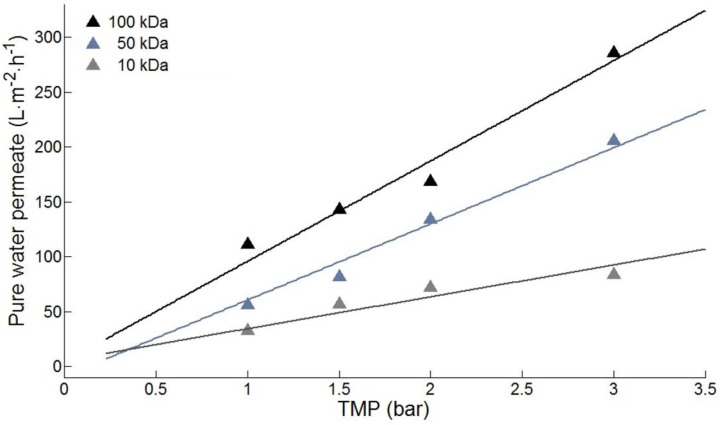
Volumetric flux as a function of transmembrane pressure for polyethersulfone (PES) membranes of different MWCO (T = 22.5 °C).

As previously mentioned, permeability tests were carried out prior to each Taguchi experiment with pure water as the feed, in order to determine the intrinsic resistance of the membrane for each membrane used, as illustrated in Figure 2.

The specific resistance values obtained from the permeability test for the membranes of 10, 50 and 100 kDa were 3.46 × 10^12^, 4.56 × 10^12^ and 9.88 × 10^12^ m^−1^, respectively.

The values for the average permeate flux, COD rejection rate and the cumulative flux decline caused by membrane fouling for each trial experiment according to the Taguchi design are shown in Table 4. The highest average permeate flux was obtained in Trial 8 (${\bar{J}}_{P}$ = 95.16 L·m^−2^·h^−1^) and the lowest value was obtained in Trial 1 (${\bar{J}}_{P}$ = 15.23 L^−1^·h^−1^). The corresponding average flux decline indices were respectively $\bar{FD}$ = 44.87% and a $\bar{FD}$ = 14.0%.

For some trials, significant differences on the results can be observed between replicates. The reason can be found in the inhomogeneous behavior between samples taken from the same sheet. The replication used in the experimental method aims to diminish the effect of the membrane variability on the response.

**Figure 3:**
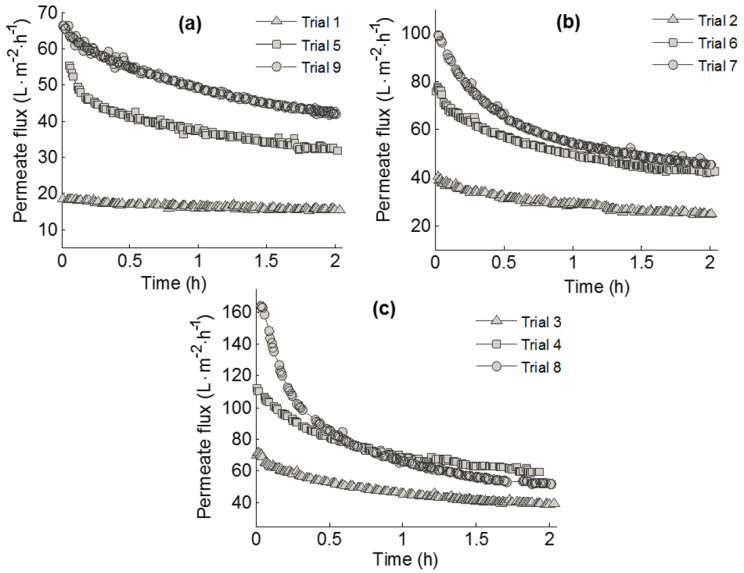
Evolution of the permeate flux through the operating time for each MWCO: (**a**) 10 kDa, (**b**) 50 kDa, and (**c**) 100 kDa (values are the average of the three replicates).

**Figure 4:**
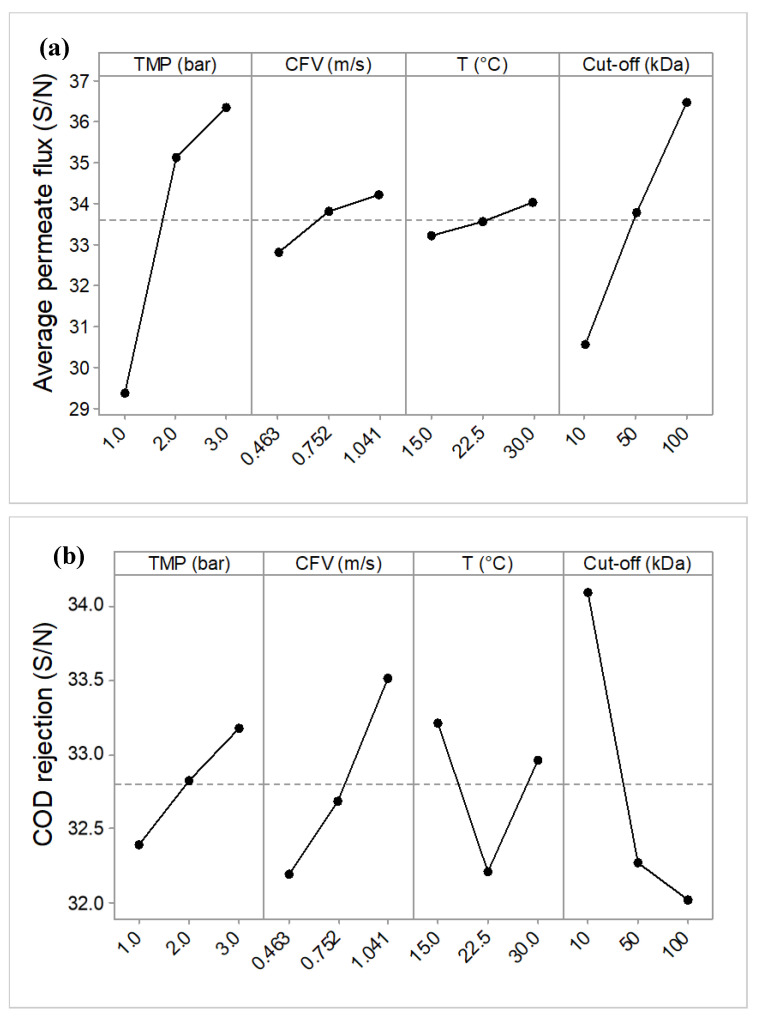

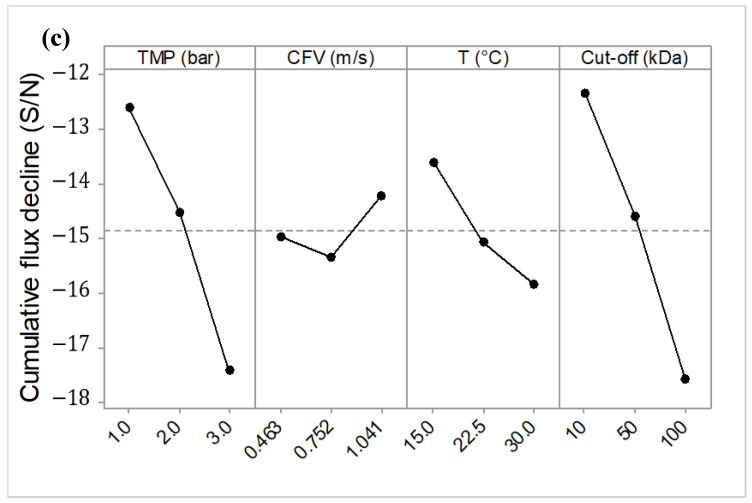
Mean effect curves for S/N ratios for (**a**) the average permeate flux, (**b**) COD rejection rate, and (**c**) the cumulative flux decline (SFD).

Figure 3 shows the evolution of the permeate average flux for all the trials carried out according the Taguchi design of Table 3. It can be seen that for the same MWCO, the permeate flux decreased with increasing TMP in all trials because of the membrane fouling. An increase in the TMP leads to higher accumulation of colloidal substances on the membrane surface and pore blocking. At the beginning of the process, flux declined very quickly, mainly for the trials corresponding to higher TMP and MWCO (Figure. 4c), possibly because of the membrane pores becoming blocked more rapidly by adsorption and the accumulation of colloidal substances. Afterwards, the permeate flux continued to decline due to the growth of a cake layer on the membrane surface, until the permeate flux reached a quasi-stationary state [53,54,55,56].

### 3.2. Taguchi Results

The corresponding S/N ratio (in dB) calculated for the response variables at each trial are listed in Table 5.

In order to analyze the influence of each factor on the response variable, the S/N ratio for a single factor can be determined by averaging the S/N ratios at their levels. The range of the effect for each factor (Δ*s*) is calculated as the difference between the two readings, the higher the range, the stronger the effect of the factor, in other words, it shows which parameter has the greatest effect on the response.

The mean S/N ratio curves for each factor are shown in Figure 4. It is worth mentioning that the peak points in these plots correspond to the optimal condition.

As can be seen in Figure 4, the variations (Δ*s*) around the mean S/N value were different for the different factors. TMP and membrane MWCO had the greatest effect on the average permeate flux as they have the steepest slope, Δ*s* = 7.10 and 5.95 respectively. CFV was the next one with a Δ*s* = 1.42, and temperature had the lowest variation around the mean S/N value, with Δ*s* = 0.78. In addition, from Table 5, the overall mean value was calculated as 33.59 (dB) from all the trial experiment results. It can be observed that the increase in ${\bar{J}}_{P}$ was stronger when the TMP changes from 1.0 to 2.0 bar than when it changes over the range from 2.0 to 3.0 bar, this could be due to the effects of polarization and cake compaction on the membrane surface. For CFV, the slope of the line between the different levels is not the same (0.463–0.752 m/s is higher than at 0.752–1.041 m/s), but with a small variation around the ${\bar{J}}_{P}$ value. Also, it can be seen for MWCO and temperature that the slopes from 10 to 100 kDa and 15 °C to 30 °C (respectively) are almost the same. Therefore, the maximum average permeate flux can be obtained for 3.0 bar, 100 kDa, 1.041 m/s CFV and high temperature (30 °C).

Under optimal COD rejection conditions, a positive and larger value of S/N is desired. In Figure 4b when comparing the S/N between different factors, it was shown that the most significant variation around the mean S/N ratios is observed for MWCO and CFV (Δ*s* = 2.07 and 1.32 respectively). Also, it can be seen that the S/N ratio increased with TMP and CFV and decreased with MWCO. Hence maximum COD removal occurred at higher TMP and CFV (3.0 bar and 1.041 m/s), and 10 kDa. It is worth mentioning that the DCS found in the PMTE are a mixture of high and low molecular weight organic and inorganic compounds, thus the contribution of the smaller particles gives lower rejection during high MWCO UF in membranes with 50 and 100 kDa MWCO.

Figure 4c shows that an increase in TMP, temperature and MWCO caused a decrease in the S/N ratio for SFD, that is to say, these factors intensified the membrane fouling effects. On the other hand, an increase in CFV induced an increase in the S/N ratio, this resulted in a decrease in the fouling effect. The highest variations around the mean S/N ratio were found for MWCO and TMP (Δ*s* = 5.21 and 4.81). Generally, the permeate flux increased with increasing MWCO and TMP. However, under these operating conditions, DCS in PMTE can easily pass through the membrane and blocking can be observed within the pores and on the membrane surface. In addition, the highest S/N ratio for the SFD factor (−8.98 ± 0.28) was achieved in Trial 1, whereas the lowest S/N ratio (−19.43 ± 0.29) was obtained in Trial 8. The optimal conditions that minimized the SFD (lowest level of fouling) were obtained at the lowest TMP (1.0 bar), highest CFV (1.041 m/s), at temperature 15 °C and at the smallest MWCO (10 kDa).

### 3.3. ANOVA Results

A statistical analysis of variance (ANOVA) was carried out to quantitatively determine the effect of each factor on the UF process indicators, with the aim of estimating whether the process parameters are statistically significant or not on the results responses. The ANOVA results are shown in Table 6.

In order to determine the qualitative significance of each factor on the responses, Fisher’s test (F-value) was employed in the ANOVA analysis. An F-value is defined as the ratio of variance due to the effect of a factor on the variance due to the inherent error in the system [57]. The F-value was compared to the critical F-value (Fcr) [52]. A calculated F-value lower than the Fcr-value means that the effect of that factor is not significant at the selected confidence level or/and it is not important in comparison with the error term. In this study, with four factors, three levels for each factor and three repetitions at each trial condition, the DOF for each factor is 2 and the DOF for the error is 18, so the Fcr-value at a confidence level of 95% is equal to 3.55. In accordance with the ANOVA table, for average permeation flux, the F-value for TMP and MWCO (114.1 and 81.52, respectively) are greater than the Fcr-value. This means that the variance of these factors is significant compared with the variance of error and they have a significant effect on the response. On the other hand, temperature and CFV had no meaningful qualitative effect on ${\bar{J}}_{P}$, as their F-values were less than the Fcr-value. Furthermore, COD rejection rate and SFD presented F-values for all factors greater than the Fcr-value which means that the effect of these factors is significant at the 95% confidence level and they have a meaningful qualitative effect on responses.

Another statistical tool that is helpful for qualitative evaluation in ANOVA is the *p*-value, which is used to indicate which factors had a significant effect on the responses. The smaller the *p*-value at an α level of significance, the more significant is the corresponding factor [58,59]. In this study, based on p-values at the 95% confidence level (α = 0.05), all the factors had a statistically significant (*p*-value < 0.05) effect on the COD rejection rate and SFD. For ${\bar{J}}_{P}$, CFV and temperature had a *p*-value higher than 0.05, thus the effect may be regarded as insignificant and it can be ignored.

**Figure 5:**
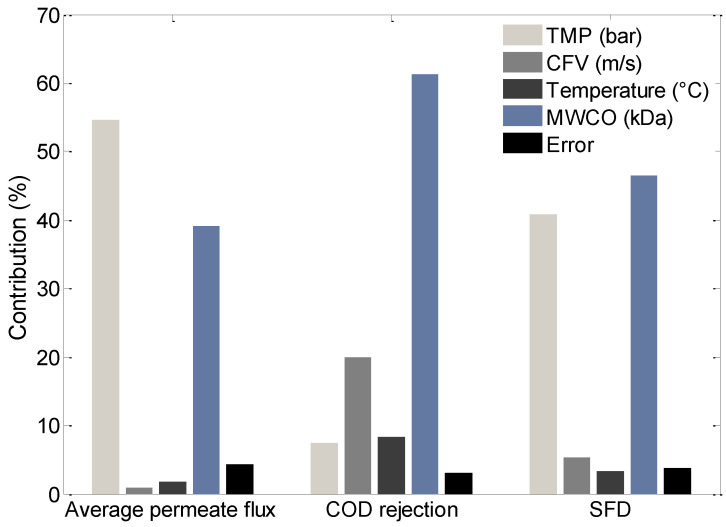
ANOVA results for the percentage contribution of each factor to the response processes.

The use of the percentage contribution (P%) in ANOVA analysis is helpful for the quantitative evaluation of the factorial effects of the performance indicators. The percentage contributions P% of all factors on average permeate flux, COD rejection rate, and SFD are shown in Figure 5. TMP (P% = 54.62) was the most important factor on average permeate flux, as higher pressure resulted in higher permeate flux, according to Darcy’s law. MWCO (P% = 39.06) was the second most important factor, followed by temperature and CFV. For the COD rejection rate, the order of importance for the factors is as follows MWCO > CFV > temperature > TMP. In addition, MWCO and TMP (46.57% and 40.89%, respectively) were the most significant parameters on membrane fouling resistance, followed by the CFV and temperature. TMP and MWCO were the most important factors for responses. Higher TMP and MWCO resulted in higher permeate flux. However, more intensive flux decline, due to membrane fouling, occurred at higher permeate flux.

It is important to mention that the values reported due to error resulting from uncontrollable noises should be below 50% for the results to be reliable [28,29,60]. Therefore, it can be seen in Figure 5 that for average permeate flux, COD rejection rate and SFD, the error values are 4.31%, 3.07%, and 3.81%, respectively. This means that the error values for the experiment are not significant for the UF process.

### 3.4. Optimal Results Obtained from the Taguchi Method and Utility Concept

The aim of optimizing the process was to find the operating conditions that led to a maximum average permeate flux and COD rejection rate based on the levels that gave the highest S/N ratios for the factors (desirable values) and to minimize the SFD, that is, the levels that gave the smallest S/N ratios (adverse values).

#### 3.4.1. Analysis of Individual Response Optimization

After identifying the optimal operating conditions, the optimal responses were predicted individually using the Taguchi method and ANOVA. Table 7 shows the Taguchi prediction results for the optimal conditions for average permeate flux, COD rejection rate and SFD.

According to the Taguchi predictions, the average permeate flux at TMP 3.0 bar, CFV 0.752 m/s, at 22.5 °C and with a 100 kDa MWCO, achieves 81.20 L·m^−2^·h^−1^. The COD rejection rate predicted under optimal conditions indicates a 57.92% rejection, higher than any value obtained in the DoE combinations. For the SFD under optimal conditions estimated by the Taguchi method, the minimum SFD predicted is approximately 1.80, equivalent to a $\bar{FD}$ of 8.65%. Therefore, we can see that the values of the three response variables combined are far from those values obtained experimentally (see Table 4).

#### 3.4.2. Analysis of Multi-Response Optimization

As mentioned above, in order to determine the weight for each response variable, a pairwise comparison matrix was compiled using the AHP method as presented in Table 8.

Thus, the weights assigned to response variables were W_JP_ = 0.568, W_COD Rejection_ = 0.098 and W_SFD_ = 0.334. The consistency ratio index (CR) is used to evaluate the consistency of AHP estimates. In this case, it was calculated as 0.021, which should be less than the allowed value of CR = 0.1, this means that the pairwise comparison matrix was considered acceptable.

The overall utility index for the *μ*_MRSN_ was calculated using Equation (13) with values associated with the weights of each response, using the lager the better (S/N) and the results are presented in Table 9.

ANOVA analysis was also performed for the multiple response variables using the utility concept. From Table 10 it is clear that when F-value is compared with Fcr (3.55), TMP, MWCO and CFV had a qualitatively significant effect (at a confidence level of 95%) on MRSN. The percentage contributions extracted from the ANOVA table were also used to determine the significance of each operating parameter in the process. The P% values were arranged as follows: TMP > MWCO > CFV > Temperature. Therefore, according to the results, TMP and MWCO were the most important factors in optimizing the multi–response UF system.

**Figure 6:**
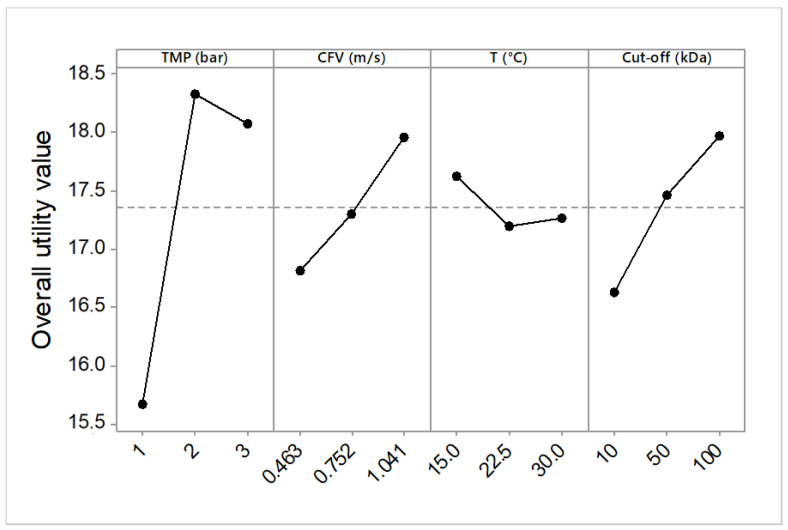
Effect of operating parameters on mean utility value (${\bar{J}}_{P}$ COD rejection rate, SFD).

The optimal operating conditions for the simultaneous response were obtained based on the criteria that both ${\bar{J}}_{P}$ and COD rejection rate must be maximized and SFD should be minimized. The variation in the overall utility for the operating parameters at different levels is presented in Figure 6.

It is clear from Figure 6 that the optimal combination of operating conditions (maximum value of the overall utility) was found at the second level of transmembrane pressure (2.0 bar), the second level of cross-flow velocity (1.041 m/s), the first level of temperature (15 °C), and third level of MWCO (100 kDa).

Once the optimal levels had been selected the next step was to estimate the multi-response S/N ratio and predict the optimal values for the simultaneous optimization response, calculated using Equation (17) and presented in Table 11.

### 3.5. Confirmation Experiment under Optimal Conditions

After determining the optimal operating conditions for the overall utility value and the significance of factors, validation experiments (for multi-responses) were carried out at the optimal levels in order to validate the predicted UF responses suggested using the Taguchi method with utility concept [34,51].

**Figure 7:**
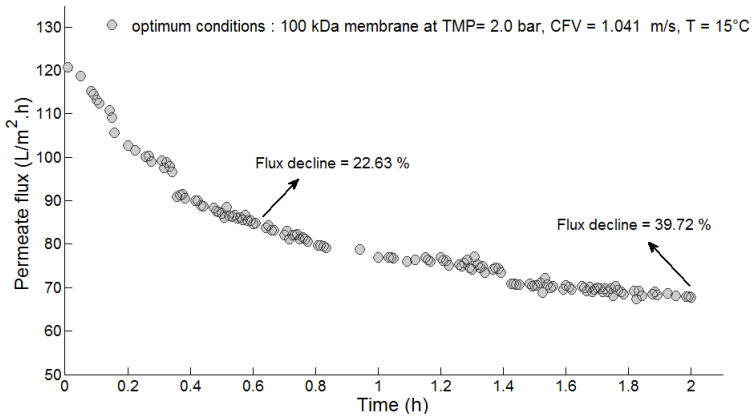
Permeate flux as a function of time under optimized conditions during UF of paper mill wastewater: PES 100 kDa membrane at TMP = 2.0 bar, CFV = 0.752 m/s and T = 15 °C.

The observed permeate flux results as a function of time under optimized conditions during the UF of PMTE are plotted in Figure 7. As described previously, the flux decline was mainly the result of two phenomena, pore blocking and cake layer formation, which mostly occurred in the first hour of the process [21,29]. During the first 30 min of the UF, the flux decreased by 22.63%. Furthermore, immediately after pore blockage, the permeate flux continued to decline due to the formation and growth of a cake layer until the system approached the quasi-steady state. At the end of the process (after 2 h), the final permeate flux was about 67.0 L·m^−2^·h^−1^ and flux decline was around 39.72%, which confirms that the membrane fouling took place with a higher rate in the first 30 min and at a slower rate when the system had achieved a steady state. Therefore, the observed experimental values of average permeate flux and cumulative flux decline were about 81.15 L·m^−2^·h^−1^ and 6.01 (SFD equivalent to a $\bar{FD}$ value of 28.96%).

The total resistance ($R_{t})$ at the end of the 2-h experiment under optimal conditions was 1.13 × 10^13^ m^−1^, which is sum of the intrinsic membrane resistance (R_m_ = 3.40 × 10^12^ m^−1^) and fouling resistance (R_f_ = 7.91 × 10^12^ m^−1^).

**Figure 8:**
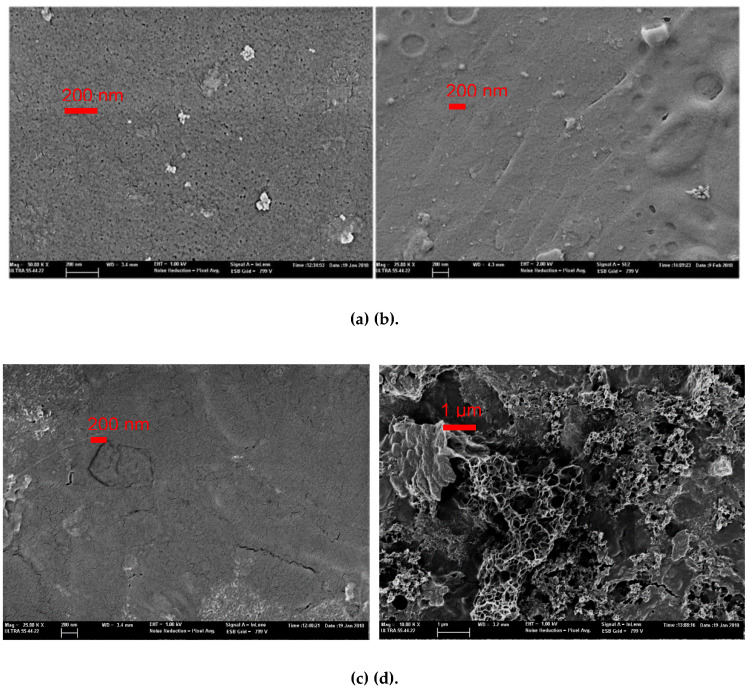
Field-emission scanning electron microscope (FESEM) image of fresh and fouled membranes (PES 100 kDa) at different operating times, (**a**) clean membrane surface, (**b**) membrane surface fouled after 30 min filtration, (**c**) at the end of the UF (2 h) with pore blocking and cake layer (**d**) membrane foulant sediments.

In addition, the membrane surface morphologies were observed by FESEM. Figure 8 shows the images of the membrane (PES 100 kDa) before and after the UF experiments were carried out. As can clearly be seen, before UF there is no blocking on the pores and no cake layer on the membrane surface. Figure 8b,c shows the surface of the membrane fouling after 30 min and after 2 h. In both cases, the images show the existence of pore blocking due to DCS adsorption within the membrane pores and sediments deposited on the surface (cake layer) acting to resist the UF [61]. Figure 8d shows the morphologies of the fouling sediments on the membrane.

Furthermore, it can be seen that the membrane was indeed fouled after 30 min filtration. However, the FESEM images of the membranes after filtration, at 2 h, were highly similar to the membranes after 30 min filtration. Therefore, it may be concluded that the permeate flux decline might result from the pore blocking as opposed to the formation and growth of the cake layer on the membrane.

Additionally, to verify the permeate applicability for paper mill reuse, the physical and chemical properties of the treated effluent obtained under optimal operational conditions (PES 100 kDa membrane at TMP = 2.0 bar, CFV = 0.752 m/s, and T = 15 °C) were compared with treated paper mil effluent used in this study as feed solution (Table 1). The results obtained for the physical-chemical parameters are given in Table 12. From the results obtained, all properties showed high retention efficiencies and proved the effectiveness of the UF under optimal conditions.

The optimum predicted results at the 95% confidence interval calculated using Equation (18) and the observed experimental results for the response variables are given in Table 13.

The observed multi-response of the overall utility falls within the 95% confidence interval for the optimal range of the response variables. In addition, it is clearly observed that the deviation between predicted and experimental results is very small, which confirms that the Taguchi method and utility concept can be used to predict the multi–response UF for any parametric combination, while individual optimization don’t got good predictions.

## 4. Conclusions

In this study, the Taguchi method, utility concept and ANOVA analysis were used as statistical tools to investigate the effects and significance of four operating parameters and to optimize the UF process with respect to average permeate flux, COD rejection rate and cumulative flux decline.

ANOVA was used to determine the most significant factors affecting the response variables. From the percentage contribution, the order of importance of each factor in maximum ${\bar{J}}_{P}$ was TMP > MWCO > T > CFV; for maximum COD rejection rate it was MWCO > CFV > T > TMP; and to achieve the minimum SFD: MWCO > TMP > CFV > T.

The optimal UF operating parameters, based on the Taguchi method and utility concept, were found at TMP (2.0 bar), CFV (1.041 m/s), temperature (15 °C) and MWCO (100 kDa). Under these optimal conditions, ${\bar{J}}_{P}$, COD rejection rate and SFD resistance of 81.15 L·m^−2^·h^−1^, 43.90% and 6.01 (around and $\bar{FD}$ value of 28.96 %), respectively, were obtained and they were within of the predicted range at the 95% confidence interval.

Measurements of turbidity, COD and particle size in the permeate showed a significant decrease 3.21 to 0.0002 NTU, 146 mg/L to 81.8 mg/L and 458–1281 nm to 12.71–24.36 nm, respectively, which confirms a substantial reduction in colloidal compounds. Therefore, it can be said that UF is suitable for removing dissolved and colloidal substances from wastewater effluents from recycled paperboard manufacturing.

Finally, we can say that the Taguchi method and utility allow membrane conditions for the P & P wastewater treatment to be optimized using a reduced number of experiments. The methodology used in this study could be used as a guideline for operating UF systems applied as a tertiary treatment for paperboard mill treated effluents under optimal conditions.

## Figures and Tables

**Table 1:** Average compositions of the ultrafiltration (UF) feed solution (prefiltered solution from a secondary clarifier effluent from a wastewater treatment plant (WWTP)).

Parameter	Units	Value
Suspended solids (TSS)	g·L^−1^	0.046 ± 0.01
Turbidity	NTU	3.21 ± 0.5
Conductivity	mS·cm^−1^	4.20 ± 1.0
COD	mg·L^−1^	146 ± 5.0
Total nitrogen	mg·L^−1^	0.8 ± 0.01
pH	−	8.30 ± 0.5

Pre-filtered by conventional filtration (pretreatment).

**Table 2:** Operating parameters and their levels.

Parameters	Labels	Levels		
		L_1_	L_2_	L_3_
TMP (bar)	A	1.0	2.0	3.0
CFV (m/s)	B	0.463	0.752	1.041
Temperature (°C)	C	15.0	22.5	30.0
MWCO (kDa)	D	10	50	100

MWCO = Molecular weight cut-off.

**Table 3:** Experimental layout using L_9_ (3^4^) orthogonal array in accordance with the Taguchi method.

Experimental Trial (*)	Levels			
	A	B	C	D
1	1	1	1	1
2	1	2	2	2
3	1	3	3	3
4	2	1	2	3
5	2	2	3	1
6	2	3	1	2
7	3	1	3	2
8	3	2	1	3
9	3	3	2	1

* All experiments were carried out in a randomized run.

**Table 4:** Experimental responses of the Taguchi orthogonal array L_9_ (34) for the three repetitions of each trial (R1, R2 and R3).

Trial n°	Factors (Parameters)				Responses								
	TMP (bar)	CFV (m/s)	T (°C)	MWCO (kDa)	${\bar{J}}_{P}\;$ (L·m^−2^·h^−1^)			COD Rejection (%)			SFD		
					R1	R2	R3	R1	R2	R3	R1	R2	R3
1	1.0	0.463	15.0	10	15.23	25.74	15.97	46.25	48.9	46.85	2.92	2.73	2.80
2	1.0	0.752	22.5	50	29.47	32.85	29.18	38.85	35.83	34.23	4.90	4.59	3.99
3	1.0	1.041	30.0	100	45.15	42.07	47.79	41.83	41.77	42.85	6.19	5.98	6.18
4	2.0	0.463	22.5	100	72.04	69.67	74.30	36.92	34.23	33.75	8.07	7.68	7.06
5	2.0	0.752	30.0	10	49.54	39.02	41.43	50.42	50.96	51.96	4.29	5.97	3.99
6	2.0	1.041	15.0	50	57.79	67.81	53.69	47.69	46.25	46.85	3.77	4.22	4.56
7	3.0	0.463	30.0	50	59.14	59.59	73.57	40.46	41.37	40.68	8.29	7.94	8.46
8	3.0	0.752	15.0	100	82.50	90.55	95.16	43.75	43.85	42.05	9.73	9.16	9.21
9	3.0	1.041	22.5	10	48.11	50.32	49.30	52.5	53.46	55.38	5.65	4.81	5.46

**Table 5:** Signal-to-noise results (mean ± standard deviation for the three repetitions at each trial).

Trial n°	S/N Ratio		
	${\bar{J}}_{P}\;$	COD Rejection	SFD
1	24.88 ± 2.52	33.50 ± 0.25	−8.98 ± 0.28
2	29.65 ± 0.57	31.16 ± 0.56	−13.13 ± 0.94
3	33.03 ± 0.55	32.49 ± 0.12	−15.73 ± 0.16
4	37.14 ± 0.28	30.85 ± 0.42	−17.64 ± 0.59
5	32.60 ± 1.09	34.17 ± 0.13	−13.67 ± 1.86
6	35.41 ± 1.04	33.43 ± 0.14	−12.46 ± 0.82
7	36.01 ± 1.08	32.22 ± 0.10	−18.31 ± 0.28
8	39.01 ± 0.67	32.71 ± 0.20	−19.43 ± 0.29
9	33.84 ± 0.19	34.61 ± 0.24	−14.52 ± 0.74

**Table 6:** Analysis of variance (ANOVA) results for average permeate flux, COD rejection, and SFD for each factor.

Responses	Factors	DOF	SS	MS	F-Value	*p*-Value
	TMP (bar)	2	6357.68	3178.84	114.01	0.000
	CFV (m/s)	2	54.76	27.38	0.98	0.394
Average permeate flux	Temperature (°C)	2	179.15	89.58	3.21	0.064
	MWCO (kDa)	2	4546.30	2273.15	81.52	0.000
	Error/others	18	501.90	27.88		
	Total	26	11,639.80			
	TMP (bar)	2	73.55	36.78	21.70	0.000
	CFV (m/s)	2	198.21	99.11	58.47	0.00
COD rejection	Temperature (°C)	2	82.58	41.29	24.36	0.00
	MWCO (kDa)	2	607.95	303.98	17.34	0.00
	Error/others	18	30.51	1.70		
	Total	26	992.79			
	TMP (bar)	2	46.48	23.24	96.57	0.000
	CFV (m/s)	2	6.12	3.06	12.72	0.000
SFD	Temperature (°C)	2	3.80	1.90	7.89	0.003
	MWCO (kDa)	2	52.95	26.47	110.00	0.000
	Error/others	18	4.33	0.24		
	Total	26	113.68			

**Table 7:** Individual Taguchi predictions for average permeate flux, COD rejection rate and SFD.

Response Variables	Optimum Operating Conditions	Significant Factors	Predicted Optimal Responses	
			S/N Ratio (dB)	Value
${\bar{J}}_{P}$	A3, B3, C3, D3	A, D	38.19	81.20 L·m^−2^·h^−1^
COD Rejection	A3, B3, C1, D1	A, B, C, D	35.26	57.92%
SFD	A1, B3, C1, D1	A, B, C, D	−5.10	1.80

**Table 8:** Pairwise comparison matrix.

Response	$\mu_{1}$	$\mu_{2}$	$\mu_{3}$
$\mu_{1}$	1.0	5.0	2.0
$\mu_{2}$	1/5	1.0	1/4
$\mu_{3}$	1/2	4.0	1.0

**Table 9:** Utility value based on UF responses (${\bar{J}}_{P},$ COD rejection, SFD) for each repetition of the experiment.

Trial n°	Utility Value		
	R1	R2	R3
1	13.60	16.42	13.96
2	15.19	15.81	15.64
3	16.69	16.43	16.99
4	18.11	18.03	18.58
5	18.37	16.24	17.72
6	19.45	19.89	18.53
7	17.14	17.32	18.17
8	18.39	19.02	19.27
9	17.46	18.16	17.32

R1, R2 and R3 is the number of repetitions of the experiment.

**Table 10:** ANOVA analysis results for multi-response UF (overall utility function).

Responses	Factors	DOF	SS	MS	F-value	*p*-value	P (%)
	TMP (bar)	2	38.85	19.43	35.50	0.000	60.93
	CFV (m/s)	2	5.84	2.92	5.34	0.015	9.16
Utility concept	Temperature (°C)	2	0.94	0.47	0.86	0.440	1.47
	MWCO (kDa)	2	8.28	4.14	7.57	0.004	12.99
	Error/others	18	9.85	0.55			15.45
	Total	26	63.76				100.00

**Table 11:** Optimal conditions for multi–response UF predicted using the utility concept.

Method	Response	Optimal Conditions	Optimal Values
Multi-response	MRSN (dB)	A2 B3 C1 D3	19.82
	${\bar{J}}_{P}$ (L·m^−2^·h^−1^)		77.22
	COD (%)		45.69
	SFD/$\bar{FD}$		6.24/30%

**Table 12:** Permeate quality (process performance) under optimum conditions, at the end of 2 h operating.

Parameter	UF Permeate Quality	Percent Removal (%)
TSS (g/L)	0.0002	99.57
Turbidity (NTU)	0.08	97.51
COD (mg/L)	81.8	43.90
Total nitrogen (mg/L)	0.53	33.75
particle size (nm)	12.76–24.36	–

**Table 13:** Summary and comparison of experimental and predicted optimal conditions for PMTE.

Method	Response	Optimal Conditions	Optimal Values	95% CI_CE_	Confirmation Experiments	% Deviation
Multi-response	MRSN (dB)	A2 * B3 * C1 D3 *	19.78	18.54 ≤ MRSN ≤ 21.08	19.78	0.27
	${\bar{J}}_{P}$ (L·m^−2^·h^−1^)		77.22	68.16 ≤ ${\bar{J}}_{P}$ ≤ 86.28	81.15	4.84
	COD (%)		45.69	43.45 ≤ COD ≤ 47.92	43.90	4.07
	SFD		6.24	5.39 ≤ SFD ≤ 7.08	6.01	3.75

* Significant at the 95% confidence interval.
